# Trioxidized cysteine and aging: a molecular binomial that extends far beyond classical proteinopathic paradigms

**DOI:** 10.18632/aging.206036

**Published:** 2024-07-25

**Authors:** José Antonio Sánchez Milán, María Mulet, Aida Serra, Xavier Gallart-Palau

**Affiliations:** 1 Biomedical Research Institute of Lleida (IRBLLEIDA) - +Pec Proteomics Research Group (+PPRG) - Neuroscience Area – University Hospital Arnau de Vilanova (HUAV), Lleida, 25198, Spain; 2 Department of Medical Basic Sciences, University of Lleida (UdL), Lleida, 25198, Spain

**Keywords:** oxidative stress, unhealthy aging, t-Cys, aging diseases, aging proteome

## Abstract

Increased oxidative stress (OS) and the disruption of the equilibrium between the production of reactive oxygen species and antioxidants are key molecular features of unhealthy aging. OS results in the formation of oxidative posttranslational modifications (PTMs), some of which involve cysteine (Cys) residues in aging proteomes, and specifically, the formation of trioxidized Cys (t-Cys), which leads to permanent protein damage. Recent findings in rodents have uncovered that irregular regulation of t-Cys residues in the aging proteome disrupts homeostatic phosphorylation signaling, resulting in alterations to proteins that are analogous to those caused by phosphorylated serine (p-Ser) residues. This work contextualizes these significant findings and discusses the implications and molecular role(s) of t-Cys in the aging proteome. Furthermore, we present novel data, validating the increase of specific t-Cys sites associated with aging in a blood-related circulating human proteome. The scope and findings included here support the hypothesis that t-Cys residues may serve as important mechanistic and biological markers, warranting further exploration in the context of unhealthy aging and age-related major diseases.

## INTRODUCTION

Aging involves intricate changes in cellular mechanisms that accumulate over time. The complex relationship between augment in reactive oxygen species (ROS) and the aging process is becoming increasingly well-understood. Oxidative stress (OS), characterized by an imbalance between oxidants and antioxidants, significantly influences the aging process at a cellular level. ROS and reactive nitrogen species (RNS) play a crucial role in causing damages to macromolecules, leading to proteinopathy and cellular senescence [1, 2]. The accumulation of oxidative damage to DNA, proteins, and lipids by chronic interactions with ROS and RNS leads to the apparition of common hallmarks of aging such as cellular senescence and mitochondrial dysfunction [3–5]. Additionally, the dysregulation of key molecular processes like autophagy, mitophagy, and the unfolded protein response contributes to cellular dysfunction with aging, increasing susceptibility to cellular damage and disease development [6].

ROS are often observed as a direct byproduct of protein damage, particularly when proteins undergo oxidative posttranslational modifications (PTMs). When cells are exposed to environmental stressors or metabolic imbalances, ROS levels can surge, leading to OS. This OS can wreak havoc on cellular components, including proteins, through a process known as protein oxidation. During protein oxidation, ROS can directly interact with amino acid residues in proteins, causing modifications such as oxidation of residues like methionine (Met) or cysteine (Cys) [7, 8].

Cys oxidation is a crucial process with significant implications in various biological contexts. Studies have shown that Cys oxidation can lead to alterations in protein structure and function [9]. This sulfur-containing amino acid is particularly susceptible to oxidation, resulting in the formation of Cys sulfenic acid (Cys-SOH), a reversible modification that serves as a signaling molecule in redox signaling pathways. However, under conditions of sustained OS, Cys-SOH can undergo further oxidation to form Cys sulfinic acid (Cys-SO_2_H) and ultimately Cys sulfonic acid (Cys-SO_3_H), collectively referred to as trioxidized Cys (t-Cys) [10].

t-Cys is a significant oxidative modification that results in irreversible protein damage, which is linked to an increase in prooxidant conditions and imbalances in ROS and RNS [10]. According to our team’s previous research and other colleagues, t-Cys has been proposed as a biomarker for OS and may potentially serve as a diagnostic indicator for diseases such as Type 2 Diabetes Mellitus [10, 11]. The effects of t-Cys on redox signaling have been explored, but the majority of research in this area has been conducted on plant organisms. Redox signaling involves modifications to transcription factors, kinases, and redox-sensitive signaling proteins, which impacts cellular functions and responses. Furthermore, the generation of ROS and RNS through purinergic signaling pathways can influence redox biology by modulating antioxidant defenses and reactive species production. Taking into account the significance of these mechanisms in mammals, it is imperative to delve deeper into the role of OS in disrupting redox signaling pathways, with the aim of gaining a more comprehensive understanding and creating novel therapeutic strategies.

The potential therapeutic advantages of Cys-rich supplements to counteract age-related declines have also been proposed based on the observation that cellular levels of this amino acid decrease with age. This is because modifications in Cys residues are thought to be directly involved in the aging process. However, the specific mechanisms through which these modifications contribute to pathological aging have remained unclear for a considerable period of time. Our research has aimed to clarify the relationship between the progression of cellular aging in mammalian organisms and the accumulation of trioxidized proteins in specific proteomes [12]. We found that oxidative damage to the tricarboxylic acid cycle (TCA) enzyme malate dehydrogenase 1 (MDH1) in the aged murine brain leads to the dysregulation of bioenergetic enzymatic activity, and in old brains, MDH1 was trioxidized at Cys137, which impacts on its function [12]. Additionally, based on the findings of other colleagues, t-Cys mediated oxidative damage to key proteins involved in energy metabolism, including MDH, contributes to decreased enzyme activities and ATP production, leading to a more oxidized neuronal environment and compromised functions [13]. The role of t-Cys in cellular proteinopathy linked to aging appears thus well-established, yet its contribution to altered cellular signaling has been somewhat overlooked.

Our recent study [14] examined the potential implications of t-Cys on impaired cellular signaling by acknowledging the biochemical similarities between these decorated residues and phosphorylated serine (p-Ser) residues in proteins. The study initially focused on the skin proteomes of aging mice to explore age-related changes in t-Cys levels and the number of t-Cys residues per protein. The results indicated a significant increase in cumulative t-Cys levels and the total number of t-Cys residues in aging and aged mice proteomes compared to young groups. Furthermore, proteome-wide analyses of t-Cys modified and unmodified counterparts uncovered that t-Cys affected, on average, 63.32% of their total. Notably, biological sex demonstrated a significant impact on the overall number of t-Cys residues present in these aged proteomes. The results also indicated a significant interaction between t-Cys and p-Ser residues, which was nearly complete for proteins with significantly differentially regulated t-Cys sites in the aged proteomes. In a similar vein, both post-translationally modified residues, while spatially remote within the primary structure of the affected proteins, were occasionally found in close proximity within the folded structure. Our research further demonstrates the capacity of t-Cys to engage with p-Ser-related enzymes, including specific kinases and 14-3-3 proteins, and to influence the structure and molecular dynamics of the protein in a manner analogous to p-Ser [14].

Although previous research, detailed in this work, has suggested that t-Cys may impact cellular signaling through redox signaling, our more recent findings in mice indicate that these residues, which are products of exacerbated OS and imbalanced cellular ROS and RNS levels, affect protein structure in a manner similar to p-Ser [14]. This discovery has critical implications for diseases, such as proteinopathy in Alzheimer’s Disease, where abnormal phosphorylation has been demonstrated to be a central core. Our findings also suggest that the interaction of these residues with phosphorylation signaling enzymes has a direct link to altering phosphorylation-mediated signaling in aging cells [14]. This new information opens up exciting research avenues that need to be investigated in the context of several diseases associated with aging. It is also imperative to assess whether the aging modulation of t-Cys residues observed in mice has any bearing on the human aging proteome. To this end, we present novel, unpublished data in this work that lends support to this hypothesis.

The importance of circulating t-Cys residues in signaling systemic OS cannot be overlooked, especially when evaluating the likelihood of premature aging in clinical populations. Furthermore, it is essential to take into account the potential role that these residues may have in specific proteins associated with certain aging-related diseases, such as neurodegenerative conditions, cardiovascular diseases, and cancer. The research presented in this work unveils novel data that demonstrates, for the first time, evidence of imbalanced t-Cys residues impacting specific proteins in platelet plasma fractions of senior adults compared to young counterparts. The implications of these findings are significant, as they highlight the potential of circulating t-Cys residues as biological markers in blood plasma for the stratification of aging conditions and diagnosis and prognosis of aging-related diseases.

The importance of these residues as potential therapeutic targets in unhealthy aging conditions, particularly within the Central Nervous System (CNS), should not be undervalued. Given the crucial identification of t-Cys sites impacting specific proteins linked to unhealthy aging conditions, which is feasible based on the novel data presented in this work, there is an immediate need to develop innovative antibodies against these modified residues. Furthermore, it is imperative to underscore the potential benefits of innovative therapeutic approaches for conditions that can impair individual autonomy as the global population ages. Although the obstacles associated with delivering drugs to the CNS are substantial, cutting-edge methodologies present promising prospects for surmounting these challenges. For instance, our research group has made significant strides in this area, and we have recently uncovered a new source of extracellular vesicles, which is considered one of the premier platforms for targeted delivery of compounds that can reach the CNS [15, 16]. To deepen our comprehension of molecular imbalances implicated in aging-related diseases and dementias, as discussed in this work thus far, we advocate that these strategic research endeavors are pivotal in the coming years. By capitalizing on existing knowledge as indicated, they should lead us onto a profoundly promising trajectory.

## MATERIALS AND METHODS

### Identification of suitable proteomics data from circulating proteomes in aging

The newly generated data in this work aims to confirm the existence of modulated t-Cys residues in the systemic human circulating proteomes as individuals age. To achieve this objective, we conducted an extensive search for publicly accessible datasets that included information on aging individuals and blood-related proteomes. After conducting a thorough screening of the available datasets, we identified the project with the identifier PXD050061 from the Proteomics Identification Database (PRIDE) [17]. The data in this project contains discovery-driven next-generation proteomics characterization of platelet rich plasma fractions from 12 volunteers divided in two groups, young and old, with an average age of 25.5 ± 2.3 in the young group and 65.8 ± 6.0 years in the old group, respectively. Further details on the data included in this project, such as collection of the samples, processing of samples for shotgun proteomics, mass spectrometry instrument acquisition parameters, etc. can be found in [17]. Briefly, blood was collected with the anticoagulant citrate and plasma was separated by centrifugation at 200 g for 10 min at 4° C. Platelet fractions were obtained by by double centrifugation at 100,000 × g for 1 hour at 4° C. Platelet proteomes were processed and digested using the commercial strategy S-Trap Micro Spin Column according to the manufacturer’s instructions (Protifi, NY, USA). The label-free LC-MS/MS analysis of platelet peptidomes was carried out using a high-throughput instrument, the Orbitrap Fusion Tribrid mass spectrometer, as described previously [18].

### Bioinformatics and data analysis

Bioinformatic analysis of the downloaded proteomics raw data was carried out, as previously indicated, by using the specialized proteomics suite software PEAKS Studio X (Bioinformatics Solutions INC., Waterloo, Canada). Precursor ion tolerance was set to 10 ppm, and fragment ion tolerance was set to 0.05 Da. Carbamidomethylation of Cys was set as fixed modifications. The human Uniprot database (downloaded on 5 September 2023, containing 207,883 protein sequences) was used for the identification of proteins. Decoy fusion, FDR < 1%, was established for protein identification in all samples, and trypsin with cleavage on at least one end was set as a proteolytic enzyme [19]. Only peptides with p-Ser and/or t-Cys modified residues and an AS score of 1000, which signifies maximum identification confidence in the PEAKS algorithm, were taken into account [20]. The results obtained from the database search were exported into comma-separated value files for subsequent analysis.

The data analysis was conducted using R software (version 4.2.1). Based on spectral count, label-free relative quantification of platelet plasma proteins between groups was performed, as previously reported [21–23]. The Brown–Forsythe test was used to assess the homogeneity of the data obtained. In cases where non-parametric analysis was required, a one-way ANOVA on ranks was performed. The level of statistical significance was set at p < 0.05, unless otherwise mentioned. Parametric one-way ANOVA with Tukey’s test for multiple comparisons was used to analyze the data further. The level of statistical significance was set at corrected p < 0.05, unless stated otherwise. Additionally, the “clusterProfiler” package (version 4.6.2) was installed in R software (version 4.2.3) to perform Gene Ontology functional analysis and pathway enrichment analysis.

### Availability of data

The data generated in this work have been thoroughly outlined in the table and graphic components. The raw data examined is accessible through the specialized repository PRIDE and can be accessed using the identifier PXD050061.

## RESULTS AND DISCUSSION

### Augmented t-Cys residues in specific circulating proteomes of aging individuals

In order to substantiate the hypothesis suggested by previous research in animal models, as stated above, regarding an augmented accumulation of t-Cys residues, which impacts specific proteins within the aging proteome and have the potential to circulate throughout the body. The recent work by Jiang et al. [17], that includes publicly available proteomics data on platelet rich plasma of individuals aged over 60 years old, spanning both young and senior age groups, was analyzed here. The aim was to determine whether any disparities in the circulating platelet proteome between young and older adults could be detected with respect to the presence of t-Cys.

**Figure 1:**
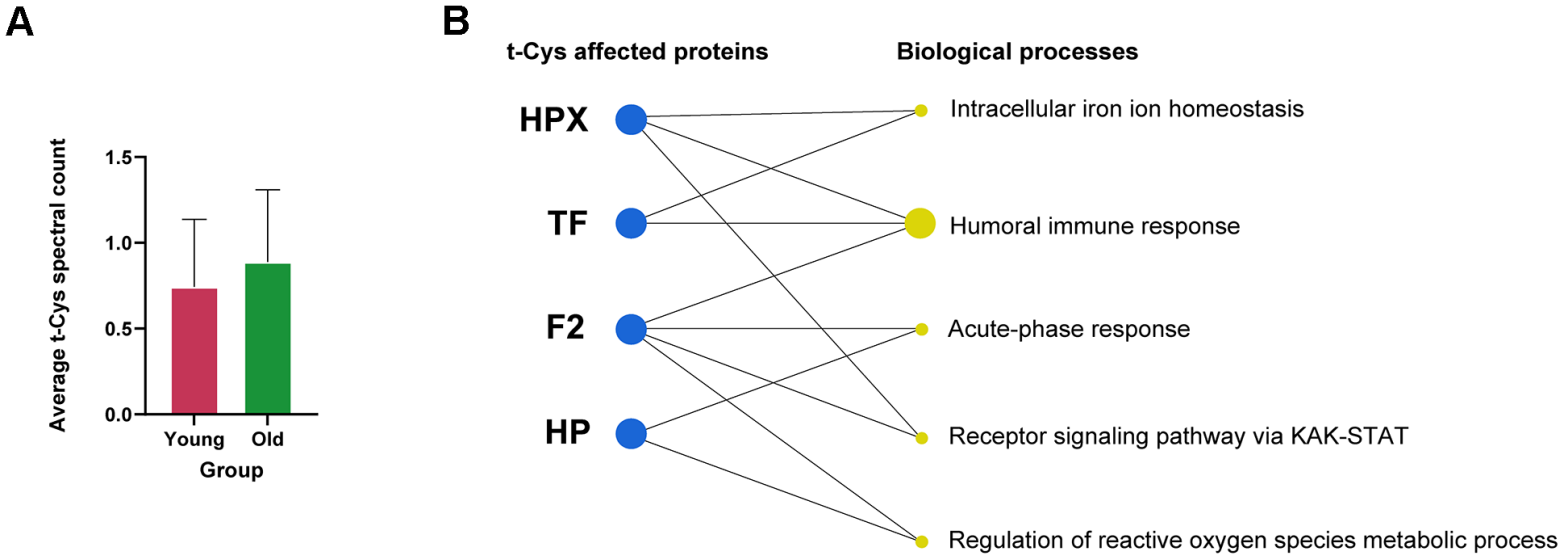
**Analysis of the levels of t-Cys residues in the circulating blood plasma platelet proteome of young and old subjects.** (**A**) Average level of t-Cys residues in proteins, categorized by age group. (**B**) Functional enrichment of differentially expressed peptides featuring t-Cys modification grouped by protein and age. Proteins exhibiting notably greater levels in older individuals as compared to their younger counterparts are depicted as blue dots. The size of the biological process dots corresponds to the differential number of t-Cys proteins incorporated. Significance was established at p<0.05, and the error bars in the graphs represent the SEM.

According to the analysis conducted, it was observed that t-Cys residues accumulated in the highly specific platelet-rich fraction of blood plasma. However, the differences between the young and old groups did not reveal substantial global significance, and the increase in the older group was only apparent, as depicted in Figure 1A. The reason for this may be attributed to the fact that all subjects in this study were in excellent health, with a maximum age of 70 years, and that the proteome of platelets constrains the scope of the whole blood plasma proteome.

Further analysis of the proteins present in the blood platelet fractions of elderly individuals revealed that a considerable number of them contained specific t-Cys sites in a significantly higher proportion compared to their younger counterparts (Table 1). These proteins comprised of hemopexin, haptoglobin, transferrin, and thrombin, as detailed in Table 1. The categorization of these proteins, which possessed specific age-modulated t-Cys sites in the blood platelet proteome, revealed that they belonged to three main groups: cellular redox mechanisms, immunity, and intracellular iron homeostasis, as illustrated in Figure 1B.

**Table 1:** List of proteins present in platelet blood plasma that exhibit differentially modulated t-Cys sites in older individuals compared to their younger counterparts.

**Peptide**	**Gene symbol**	**Average area ± SEM**			**logFC**	**Significant**
		**Young**		**Old**		
K.LPECEADDGCPKPPEIAHGYVEHSVR.Y	HP	-		11249666,67 ± 7557543,25	9.3023	*
K.SLGPNSCSANGPGLYLIHGPNLYCYSDVEKLNAAK.A	HPX	-		410016,67 ± 410016,67	8.0775	*
R.CSPHLVLSALTSDNHGATYAFSGTHYWR.L	HPX	-		315683,33 ± 315683,33	7.9249	*
R.EGTCPEAPTDECKPVKWCALSHHER.L	TF	-		325226,33 ± 306225,17	8.7959	*
R.LAVTTHGLPCLAWASAQAK.A	F2	-		858916,67 ± 858916,67	8.5091	*
R.SAGWNIPIGLLYCDLPEPR.K	TF	-		652000 ± 652000	8.3493	*

Differentially modfied t-Cys residues are indicated in augmented bold within the identified peptide sequence. Statistical differences were assessed by differential expression analysis using EdgeR package and *significance was established at p<0.05.

As a concluding remark, previous studies in rodents have suggested the aging-related modulation of t-Cys sites in the human proteome, and this novel data confirms this notion. Furthermore, the differential circulation capacity of abnormally regulated t-Cys sites in the blood proteome, which is linked to the aging process, has been identified. This paves the way for future research aimed at understanding the implications of aging-modulated t-Cys sites in diverse human proteomes, and the potential diagnostic and prognostic value of these sites in the blood plasma proteome as it relates to unhealthy aging.
